# Neuropsychiatric Manifestations of Degenerative Cerebellar Ataxia

**DOI:** 10.3390/brainsci14101003

**Published:** 2024-10-02

**Authors:** Olivera Tamaš, Milutin Kostić, Gorica Marić, Andona Milovanović, Mladen Janković, Biljana Salak Ðokić, Tatjana Pekmezović, Nataša Dragašević-Mišković

**Affiliations:** 1 Neurology Clinic, University Clinical Centre of Serbia, Faculty of Medicine, University of Belgrade, 11000 Belgrade, Serbia; andona8@gmail.com (A.M.); jankovic95@live.com (M.J.); bsalak@gmail.com (B.S.Đ.); ntdragasevic@gmail.com (N.D.-M.); 2 Institute of Mental Health, Faculty of Medicine, University of Belgrade, 11000 Belgrade, Serbia; milutin.kostic@imh.org.rs; 3 Institute of Epidemiology, Faculty of Medicine, University of Belgrade, 11000 Belgrade, Serbia; goricamaric87@gmail.com (G.M.); pekmezovic@sezampro.rs (T.P.)

**Keywords:** degenerative cerebellar ataxias, depression, anxiety, apathy

## Abstract

Background/Objectives: Degenerative cerebellar ataxias (DCA) present a group of complex neurological disorders primarily affecting the cerebellum and its pathways. Classic manifestations include motor symptoms of cerebellar ataxia. However, emerging evidence suggests that the cerebellum also plays a crucial role in various cognitive and emotional processes. The objective was to assess the psychiatric profile of a heterogeneous group of patients with degenerative cerebellar ataxia. Methods: Our sample comprised 107 participants diagnosed with cerebellar degenerative ataxia. All patients were clinically evaluated using SARA, INAS, and different neuropsychiatric scales (ACE-R, HAMA, HAMD, AS, and GAF). Results: The majority of patients had autosomal dominant ataxia (38.3%) followed by sporadic ataxia (32.7%) with an average age at the moment of diagnosis of 35.3 ± 16.23 years, while the mean duration of disease at the study beginning was 12.1 ± 9.9 years. Psychiatric disorders were present in 40 patients (37.4%), with dysthymia (14.2%), major depressive disorder (9.4%), and MDD with melancholic features (7.6%). The presence of MDD with melancholic features was statistically significantly correlated with a lower ACE-R total score (r = −0.223; *p* = 0.022), while dysthymia was statistically significantly associated with a shorter duration of the disease (r = −0.226; *p* = 0.020) and older age (r = 0.197; *p* = 0.043). Statistically significant differences were observed between MSA-C patients and those with sporadic ataxia (HDRS *p* < 0.001, HARS *p* < 0.001, Apathy Scale *p* = 0.003, and GAF *p* = 0.004). Conclusions: Based on our findings, we can conclude that the degree of motor deficit has a significant impact on the development of psychiatric disorders, including depression, anxiety, and apathy. However, it is not the only factor, and the impact also depends on the type of DCA.

## 1. Introduction

Degenerative cerebellar ataxias (DCA) are a group of complex neurological disorders primarily affecting the cerebellum and its pathways. Main clinical manifestations include impairments in coordination, balance, and motor control [1]. Traditionally, research has predominantly focused on the motor symptoms of cerebellar ataxia, while psychiatric manifestations have been relatively underexplored. However, emerging evidence suggests that the cerebellum plays a crucial role not only in motor functions but also in various cognitive and emotional processes [2,3,4,5,6,7]. This dual functionality raises important questions about the neuropsychiatric dimensions of cerebellar ataxia, particularly in the context of the neurodegenerative forms of the disorder.

Recent studies have begun to shed light on the neurobiological mechanisms underlying psychiatric symptoms in cerebellar ataxia. For instance, disruptions in cerebellar-cortical networks have been implicated in the pathophysiology of mood disorders associated with ataxia [8,9]. Additionally, neuroimaging studies have demonstrated structural and functional changes in the cerebellum and its connections that correlate with psychiatric symptoms [10]. Understanding these relationships is critical for developing comprehensive treatment approaches that address both the neurological and psychiatric aspects of the disease.

Degenerative cerebellar ataxias, including conditions such as spinocerebellar ataxias (SCAs), multiple system atrophy-cerebellar type (MSA-C), etc., are marked by progressive cerebellar degeneration. As these diseases advance, patients often experience a range of psychiatric symptoms, including depression, anxiety, and even psychosis [6,11]. These psychiatric manifestations can significantly impact the quality of life [12], complicating disease management and presenting challenges for both clinicians and caregivers [6].

Given the increasing recognition of the cerebellum’s role in mental and behavioral disorders, our study aimed to assess the psychiatric profile in a heterogeneous group of patients with degenerative cerebellar ataxia.

## 2. Methodology

### 2.1. Materials and Methods

#### 2.1.1. Study Design

Before study initiation, ethical approval was obtained from the Ethical Committee of the Faculty of Medicine, University of Belgrade. All participants consented to take part in this study by signing an informed consent. Previous research papers have already provided a detailed description of the current study [12,13]. In summary, our cross-sectional study included 107 (48 males and 59 females) consecutive degenerative cerebellar ataxias patients aged 18 or older who reported to the Neurology Clinic of the University Clinical Center of Serbia between 2017 and 2020.

The patient’s average age was 47.5 ± 12.4 years. Patients’ age at the time of diagnosis was, on average, 35.3 ± 16.23 years, while the mean duration of disease was 12.1 ± 9.9 years. In terms of clinical characteristics, patients’ average scores on the Scale for Assessment and Rating of Ataxia (SARA), Inventory of Non-Ataxia Signs (INAS), and Addenbrooke’s scale for assessing cognitive function—revised version (ACE-R) scales were 14.9 ± 7.0 (range 2–40), 4.5 ± 1.9 (1–9) and 79.1 ± 15.8 (35–100), respectively. Out of the total sample (n = 107), the majority of patients had autosomal-dominant ataxia (AD) (38.3%), followed by sporadic adult-onset ataxia (SAOA) (32.7%), autosomal recessive ataxia (AR) (19.6%), and MSAc (9.3%).

In the AD group (n = 41), 12 patients were suffering from SCA1, seven participants had SCA2, and only one patient was diagnosed with SCA7. In another 21 participants, genetic findings were categorized as undetermined, but their clinical presentation corresponded to an apparently autosomal dominant pattern.

In the AR group of patients (n = 21), AR spastic ataxia of Charlevoix-Saguenay (ARSACS) was found in one participant, ataxia with oculomotor apraxia type 2 (AOA2) also in one patient, three patients were positive for biallelic repeat expansion in the *RFC1* gene (i.e., they were CANVAS patients), four patients had an apparently AR inheritance pattern that was genetically undetermined, five had biallelic repeat expansion in *FXN* gene (i.e., they were affected by FRDA), and seven had homozygous pathogenic variant in *ANO10* gene.

Sporadic adult-onset ataxia (SAOA) was diagnosed in thirty-five patients (n = 35), and ten diagnosed with multiple system atrophy-cerebellar subtype (MSA-C) were recruited as well (n = 10).

Patients who had proven other causes of cerebellar ataxia (metabolic, endocrinological, paraneoplastic, or immunological), patients with known chronic consumption of alcohol, drugs, or toxic substances that can cause ataxia, or in case they met the criteria for establishing another neurodegenerative disease were excluded.

At the time of study participation, a certain number of included patients (n = 20) were treated with antidepressants and/or other psychiatric medications (e.g., sertraline, fluoksetin, clonazepam, and alprazolam). None of the patients received psychotherapy.

The information was extracted from the patient’s medical records and complemented with findings obtained during clinical examination provided by an experienced neurologist.

#### 2.1.2. Instruments

This study relied on the use of several scales and one neuropsychiatric questionnaire. While a number of them have been described elsewhere [12,13], some are detailed in the current paper. The severity of ataxia was revealed with the SARA [14]. To evaluate the non-cerebellar signs, we used the INAS [15]. ACE-R was used to evaluate the global cognitive function of participants [16].

##### M.I.N.I. International Neuropsychiatric Questionnaire

This structured diagnostic interview is designed to assess a wide range of psychiatric disorders. According to criteria from the DSM (Diagnostic and Statistical Manual of Mental Disorders) included are mood disorders (depression, bipolar disorder), anxiety disorders (panic disorder, social phobia), psychotic disorders, and substance use disorders [17].

##### Assessment of Anxiety

To assess the level of anxiety in our patients, we used the Hamilton Anxiety Rating Scale (HAMA) [18]. A trained physician completes this scale based on the interview carried out with each patient individually. The scale scores 14 symptoms from 0 (not present) to four (severe), resulting in a total score ranging between 0 and 56. Patients who score 13 points or more are considered anxious.

##### Assessment of Depression

To assess depression, we used the Hamilton Depression Rating Scale (HAMD) [19], where a trained physician conducts a structured interview with the participants. A score above 17 is considered to indicate depression.

##### Assessment of Apathy

The Modified Scale to Assess Apathy (AS, from English Apathy Score) [20], which contains 14 questions, was used to determine apathy. Patients with ≥14 points were considered apathetic.

##### Assessment of Functionality

The Global Assessment of Functionality (GAF) scale [21] is an instrument that medical professionals use to assess patients’ social, professional, and psychological functioning in individual daily activities. It is a numerical scale with scores ranging between 0 and 100, where high values mean an individual has minimum difficulties performing everyday functions, while values of 55 or less indicate serious difficulties and potential danger to oneself or others.

##### Statistical Analysis

Descriptive data analysis included calculating mean values accompanied by standard deviation for continuous variables and frequencies with percentages for categorical data. Differences in scale scores between compared groups were determined using one-way ANOVA, while the post-hoc analysis was performed using Tukey’s test. Relationship between variables was established with Pearson and Spearman correlation analysis. Data analysis was conducted using SPSS (Statistical Package for Social Sciences), version 20.0.

## 3. Results

**Figure 1:**
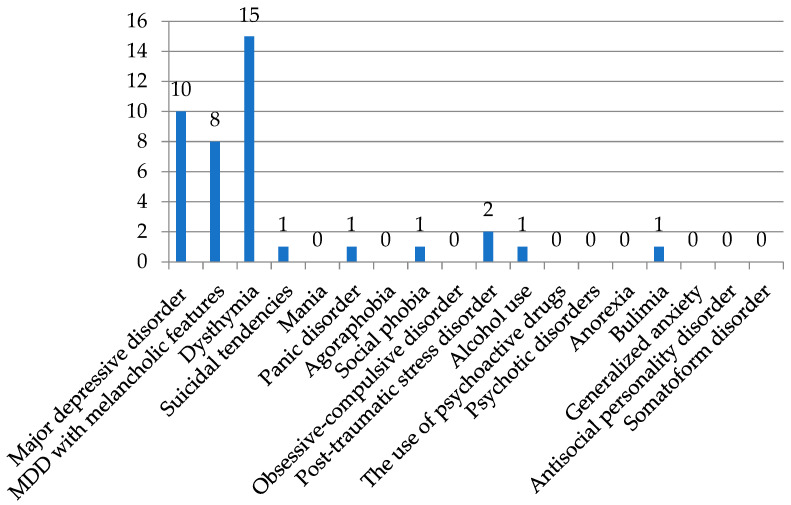
Distribution of different psychiatric disorders estimated by M.I.N.I. questionnaire in DCA spectrum patients.

Out of 107 patients with DCA spectrum disorders, 40 (37.4%) patients had some psychiatric disorder. The most prevalent were dysthymia (14.2%), major depressive disorder (MDD) (9.4%), and MDD with melancholic features (7.6%). Post-traumatic stress disorder was detected in two patients (1.9%), while suicidal tendencies, social phobia, alcohol abuse, and bulimia were noted in individual cases. Other psychiatric disorders were not present (Table 1 and Figure 1).

An examination of the association of the presence of psychiatric disorders with different demographic and clinical characteristics of the test subjects showed that the presence of MDD with melancholic features was statistically significantly correlated with a lower ACE total score (r = −0.223; *p* = 0.022). The presence of dysthymia was statistically significantly correlated with a shorter duration of the disease (r = −0.226; *p* = 0.020) and older age (r = 0.197; *p* = 0.043). The presence of bulimia was significantly associated with a longer duration of the disease (r = 0.306; *p* = 0.001) (data is not displayed).

**Figure 2:**
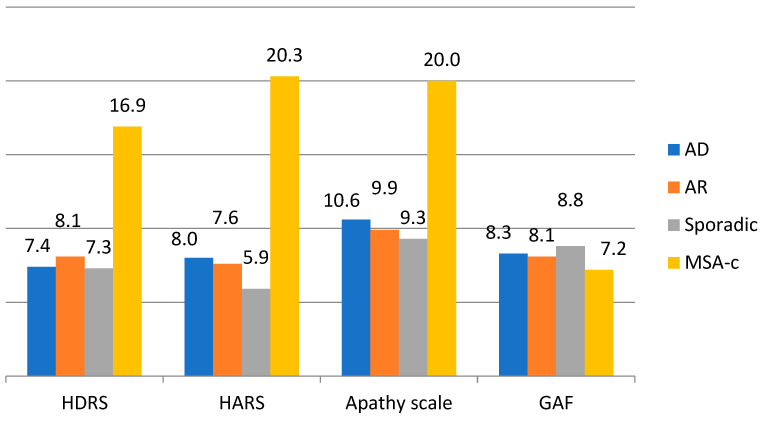
Distribution of psychiatric scale scores according to the DCA type.

Table 2 and Figure 2 present the average scores of the psychiatric tests used in our study for the entire group of subjects and each patient subgroup. The average score in the entire study population on the HDRS was 8.4 ± 6.0; on the HARS, it was 8.4 ± 6.8; on the Apathy Scale, it was 10.9 ± 8.3, while on the GAF scale, it was 8.3 ± 1.3. Patients with MSA-C had the highest average scores on the HDRS (16.9 ± 1.0), HARS (20.3 ± 2.8), and Apathy Scale (20.0 ± 5.1), while patients with SAOA had the highest average score on the GAF scale (8.80 ± 1.45). Statistically significant differences were observed only between MSA-C patients and those with SAOA (HDRS *p* < 0.001, HARS *p* < 0.001, Apathy Scale *p* = 0.003, and GAF *p* = 0.004).

Table 3 shows the correlation between the scores of various psychiatric tests and the demographic and clinical characteristics of the respondents. The HDRS score was statistically significantly correlated with age (r = 0.243, *p* < 0.05), ACE-R score (r = −0.266, *p* < 0.01), and SARA score (r = 0.349, *p* < 0.01). The HARS score was significantly associated with age (r = 0.237, *p* < 0.05) and SARA score (r = 0.214, *p* < 0.01). Further, the Apathy Scale score correlated with the ACE-R score (r = −0.288, *p* < 0.01), SARA score (r = 0.42, *p* < 0.01), and INAS score (r = 0.213, *p* < 0.05), while the GAF scale score was significantly correlated with the duration of the disease (r = −0.248, *p* < 0.01), ACE-R score (r = 0.383, *p* < 0.01), SARA score (r = −0.439, *p* < 0.01), and INAS score (r = −0.345, *p* < 0.01).

## 4. Discussion

The incidence of psychiatric disorders, as assessed by the MINI questionnaire in our study, was 37.6%, of which the most prevalent were dysthymia (14.1%), MDD (9.4%), and MDD with melancholic features (7.6%). Leroi et al. [22] have shown that the incidence of mood disorders is significantly higher in patients with neurodegenerative ataxia compared to our results. In their study, 35.5% of patients have met the criteria for major depression at some point in their illness, while 32.2% of patients have met the criteria for non-major depression. This difference in the incidence of mood disorders can be explained primarily by a different methodology since our results were related to the psychiatric status of patients at the time of testing. Furthermore, in the group of patients with non-major depression described in Leroi et al., patients with minor depression and recurrent depressive disorder are included in addition to the patients with dysthymia. Much closer results to ours were obtained by Lizsewski et al. [3]. In their study, 41% of DCA patients had some psychiatric disorder, with depression emerging as the most prevalent condition.

In addition to categorizing patients according to the DSM-IV classification using the M.I.N.I questionaries, different scales were used in our study to assess the degree of severity of psychiatric symptoms. The average HADS scores in our patients were higher (8.4 ± 6.0; 8.4 ± 6.8; vs 5.35 + 4.54, 5.07 + 4.59) than those in the study of Leroi et al. [22]. This difference in the results of depression can be partly explained by the difference in patient composition since our heterogeneous group of patients also included those with AR ataxias. According to the literature, the incidence of depressive episodes in these patients ranges up to 50% [7].

Emerging evidence suggests that the posterior vermis, also called the limbic cerebellum, modulates emotional processing through its anatomical and functional connections with limbic structures like the amygdala and hippocampus. Lesions and disruptions in these cerebellar regions are linked to various psychiatric and mood disorders, including depression [9,23]. Some studies have reported smaller cerebellar volumes in patients with depression and bipolar disorder [24], while Yucel et al. found significantly reduced vermis volume in individuals with MDD [25]. Further evidence supporting the hypothesis that vermal regions modulate limbic activity includes findings that patients with strokes in these regions exhibit increased PET activity in the prefrontal cortex and decreased activation in the limbic system in response to unpleasant stimuli [26].

By far, the most pronounced symptoms of depression, anxiety, and apathy in our patients were those diagnosed with MSA-C compared to other subgroups of patients. Depression is one of the most prominent non-motor symptoms in multiple systemic atrophy. The prevalence of depression is over 50% in both motor subtypes of MSA [11,27]. It is believed that the cause is multifactorial and that, in addition to the influence of disability, the neurodegeneration of noradrenergic and cholinergic neurons contributes to the appearance of this disorder [28]. It was observed that patients in the AD ataxia group had a lower mean score on HDRS (7.4 ± 5.1) compared to the MSA and AR subgroups of patients and in the level of sporadic ataxias. The prevalence of depressive symptoms in patients with SCA ranges between 17 and 26%, and the frequency varies depending on the type of SCA [29,30]. SCA3 is the phenotype with the most common depressive symptoms and a degree of suicidal ideation [30]. The absence of this phenotype in our study may explain a low score on the HDRS scale. According to the results of Abele et al., the incidence of moderate and severe depression in SAOA patients ranges around 38% [31].

Several potential factors could explain such differences in depression rate and severity between MSA-C and other types of DCA. The severe and rapidly progressive nature of motor and autonomic dysfunction in MSA-C, along with the resultant disability, contributes to psychosocial stress and a diminished quality of life, further predisposing these patients to depression [32,33]. Additionally, patients with MSA experience widespread neurodegeneration that extends beyond the cerebellum to involve the basal ganglia, brainstem, and autonomic nervous system, all of which are crucial for mood regulation. The characteristic pathology includes severe gliosis, neuronal loss in the pontine nuclei, and significant involvement of the olivopontocerebellar tracts [34]. On the other hand, in SCA patients, the degeneration is more restricted to the cerebellum and its afferent and efferent connections, with varying degrees of involvement of the pons and olives depending on the specific SCA subtype [35,36].

The highest level of anxiety assessed by the HARS scale also belonged to a subgroup of patients with MSA-C. It may also be due to the different prevalence of this psychiatric disorder depending on the type of DCA. According to the study conducted by Zhang et al. [37], in which an identical scale was used, 46.8% of patients with MSA had mild anxiety, and 24.9% of patients had severe anxiety. The incidence of this disorder in patients with SCA ranges from 13 to 66%; however, this disorder is not typical in phenotype SCA 1 and SCA 2, the dominant populations in our study [38]. In contrast, patients with Friedreich’s Ataxia, which belongs to ARCA, did not have an increased incidence of anxiety compared to the healthy population [38].

In addition to depression and anxiety, the highest degree of apathy assessed by the Hamilton scale also belonged to the group of patients with MSA-C. Although this symptom is associated with a disorder in the fronto-subcortical circuits (anterior cingulate cortex, anterior medial orbitofrontal cortex, and striatum), it has also been shown that isolated cerebellar lesions may be the cause [39]. According to the study of Cao et al. [40], this disorder is noted in 43.6% of patients with MSA, and Santangelo et al. [11] did not show any significant difference in the severity of this symptom between the MSA-C and MSA-P phenotypes. From the point of view of other types of DCA, apathy has not often been a subject of interest in the literature. SCA 1 and SCA 2 have shown a significantly higher incidence of apathy compared to the healthy population [41], while studies examining the presence of this disorder in patients with Friedreich’s ataxia have not been published so far.

The lowest values on the global functioning scale were expected to be attributed to patients in the MSA-C subgroup, given the prevalence and severity of psychiatric symptoms. Low scores in other subgroups of DCA should not be overlooked. In the study by Lasek et al. [10], which estimated the correlation between atrophy of certain brain regions and GAF values in patients with SCA17, lower values correlated statistically significantly with the degree of cerebellar degeneration, among other things. This further supports the evidence that primary cerebellar degeneration in some DCA patients significantly impacts the prefrontal cortex, given the well-established anatomical and functional connections between these structures [42].

In our study, the age of patients stood out as a demographic characteristic that correlated with the degree of depression and anxiety. Older patients with DCA were more depressed and anxious. Namely, one of the hypotheses is that older patients who have experienced life without disease and have realized themselves as healthy people in different fields have a harder time adapting to the newly created disability and the burden that the disease brings. All of this can lead to increased anxiety and depression [43]. Additionally, our patients with superior cognitive performance, as measured by the ACE-R, exhibited greater depression and apathy, potentially attributable to their increased awareness of the disease’s impact on their quality of life.

The severity of ataxia, as assessed by the SARA score, was statistically significantly correlated with the severity of symptoms of depression, anxiety, and apathy. Among other things, the severity of ataxia correlated with the degree of impairment of global functioning due to the presence of psychiatric symptoms in our patients. The results of other studies that have analyzed this correlation are varied and depend on the type of DCA. While in some cases, this connection has been confirmed, such as in the case of Friedreich’s Ataxia and depression [44], in patients with SCA, although depression progresses with disease progression, it is not necessarily associated with the degree of motor deficit [30,45]. It has also been shown that the degree of apathy is positively correlated with the degree of motor deficit in the SCA1 and SCA2 populations [41]. In our study, patients with more frequent non-cerebellar signs, as measured by the INAS score, showed significantly higher levels of apathy. This may be due to the burden of disease symptoms, but also the presence of extracerebellar signs indicates the involvement of extracerebellar brain structures, including the fronto-subcortical circuits, which are linked to the pathogenesis of apathy [39,46].

Finally, we would like to look at some of the limitations of this study. The study population is heterogeneous, composed of genetically mediated ataxia with different types of inheritance and sporadic neurodegenerative ataxias, which can potentially impact the results obtained. Future studies that would focus on certain phenotypes of DCA ataxia could potentially indicate a clear diversity in the prevalence of certain psychiatric symptoms and behavioral patterns about the etiology of ataxia itself. Another limitation is that the Cerebellar Cognitive-Affective Syndrome Scale, whose structure is based on the main features of this syndrome arising within the framework of damage and degeneration of the cerebellum, was not used in this study [47]. Using this scale could particularly highlight the role of the cerebellum in the production of neuropsychiatric symptoms in different types of DCA.

## 5. Conclusions

Among all psychiatric disorders, we can distinguish depression as the most common in patients with DCA. Compared to other subgroups of patients, MSAc patients had the most prominent psychiatric symptoms, probably due to the rapidly progressive course of the disease, along with the extent of neurodegeneration. Based on our findings and the results of previously mentioned studies, we can conclude that the degree of motor deficit has a significant impact on the development of psychiatric disorders, including depression, anxiety, and apathy. However, it is not the only factor; the development of psychiatric disorders also depends on the type of DCA.

## Figures and Tables

**Table 1:** Prevalence of psychiatric disorders estimated by M.I.N.I. questionnaire in DCA spectrum patients (%).

Psychiatric Disorder	Frequency of Patients with the Disorder	Prevalence of the Disorder among Patients
Major depressive disorder (MDD)	10	9.4%
MDD with melancholic features	8	7.6%
Dysthymia	15	14.2%
Suicidal tendencies	1	0.9%
Mania	0	0.0%
Panic disorder	1	0.9%
Agoraphobia	0	0.0%
Social phobia	1	0.9%
Obsessive-compulsive disorder	0	0.0%
Post-traumatic stress disorder	2	1.9%
Alcohol use	1	0.9%
The use of psychoactive drugs	0	0.0%
Psychotic disorders	0	0.0%
Anorexia	0	0.0%
Bulimia	1	0.9%
Generalized anxiety	0	0.0%
Antisocial personality disorder	0	0.0%
Somatoform disorder	0	0.0%

M.I.N.I. International Neuropsychiatric Questionnaire.

**Table 2:** Psychiatric characteristics of test subjects according to the type of ataxia.

	AD (n = 41)	AR (n = 21)	Sporadic (n = 35)	MSA-C (n = 10)	Total	*p*
	$\bar{x}$ ± SD	$\bar{x}$ ± SD	$\bar{x}$ ± SD	$\bar{x}$ ± SD	$\bar{x}$ ± SD	M
HDRS	7.4 ± 5.1	8.1 ± 5.0	7.3 ± 6.5	16.9 ± 1.0	8.4 ± 6.0	**<0.001 ^a^**
HARS	8.0 ± 5.9	7.6 ± 5.2	5.9 ± 5.9	20.3 ± 2.8	8.4 ± 6.8	**<0.001 ^a^**
Apathy scale	10.6 ± 8.5	9.9 ± 6.1	9.3 ± 8.7	20.0 ± 5.1	10.9 ± 8.3	**0.003 ^a^**
GAF	8.3 ± 1.2	8.1 ± 1.1	8.80 ± 1.45	7.20 ± 0.9	8.3 ± 1.3	**0.004 ^a^**

AD = autosomal dominant ataxia, AR = autosomal recessive ataxia, MSA-C = multiple systemic atrophy-cerebellar type, $\bar{x}$ = arithmetic mean, SD = standard deviation, HDRS = Hamilton Depression Scale, HARS = Hamilton Anxiety Scale, GAF = Global Functionality Assessment Scale, ^a^—significant difference between sporadic ataxias and MSA-C.

**Table 3:** Correlation of psychiatric characteristics on estimated (HDRS, HARS, Apathy Scale, GAF, and NPI) scales with demographic and clinical characteristics of the test subjects (age, disease duration, ACE, SARA, and INAS) for all groups of patients (n = 107).

	Age	Disease Duration	ACE-R	SARA	INAS
HDRS	0.243 *	0.085	−0.266 **	0.349 **	−0.320
HARS	0.237 *	0.039	−0.135	0.214 *	−0.266
Apathy Scale	0.173	0.153	−0.288 **	0.420 **	0.213 *
GAF	−0.089	0.248 **	0.383 **	−0.439 **	−0.345 **

ACE-R = Addenbrooke’s Cognitive Examination-Revised SARA = Scale for the Assessment and Rating of Ataxia, INAS = Inventory of Non-Ataxia Signs, GAF = Global Assessment of Functioning scale. The table contains significant correlations (correlation coefficient r/level of significance * *p* < 0.05, ** *p* < 0.01.

## Data Availability

The data from this study are available on request from the corresponding author. The data are not publicly available due to privacy and ethical restrictions.
